# Acute Toxic Effects of the New Psychoactive Substance “Voodoo” among Patients presented to the Poison Control Center of Ain Shams University Hospitals (PCC-ASUH), Egypt, during 2017

**DOI:** 10.1186/s13011-021-00408-4

**Published:** 2021-09-20

**Authors:** Rania Hussien, Maged El-Setouhy, Mohamed El Shinawi, Hazem Mohamed El-Hariri, Jon Mark Hirshon

**Affiliations:** 1 Department of Forensic Medicine and Clinical Toxicology, Faculty of Medicine, Ain Shams University, Cairo, Egypt; 2 Department of Family and Community Medicine, Faculty of Medicine, Jazan University, Jazan, Saudi Arabia; 3 Department of Community, Environmental and Occupational Medicine, Faculty of Medicine, Ain Shams University, Cairo, Egypt; 4 Department of Emergency Medicine, University of Maryland School of Medicine, Baltimore, Maryland USA; 5 Department of Surgery, Faculty of Medicine, Ain Shams University, Cairo, Egypt; 6 Department of Community Medicine, National Research Centre, Cairo, Egypt

**Keywords:** New psychoactive substances, Voodoo, Toxicity, Egypt

## Abstract

**Background:**

Voodoo is a heterogeneous mixture of psychoactive substances that has recently grown in popularity among youth in Egypt. Patients can present with a variety of manifestations that may lead to death in some cases. This study assessed the acute toxic effects of voodoo among patients presented to the Poison Control Center of Ain Shams University Hospitals (PCC-ASUH) during a one year period.

**Methods:**

This is a retrospective study of all patients presented with voodoo intoxication at the PCC-ASUH from 1 January 2017 to 31 December 2017. Clinical data, routine laboratory findings, and ECG results as well as duration of hospitalization and outcome were compiled from hospital records.

**Results:**

Seventy-one voodoo intoxication cases meeting the inclusion criteria were analyzed (mean age: 25.19 ± 9.54 years, range: 15–50 years, 97.2 % male). Pulse, blood pressure, and respiratory rate were normal in more than half of all patients. Neurological abnormalities including agitation, hallucinations, disturbance of consciousness were the most frequent manifestations. Respiratory acidosis was the most common laboratory finding (54.9 %), followed by increased serum urea (43.6 %), hypokalemia (33.8 %), hyperglycemia (28.1 %), and leukocytosis (26.7 %). The most common ECG finding was sinus tachycardia (31 %), followed by QT prolongation (15.4 %). More than half of the studied patients (53.5 %) co-administered other illicit substances, most frequently cannabis and tramadol. Most patients recovered fully and were discharged, but death occurred in two cases.

**Conclusions:**

Voodoo toxicity can manifest with many presentations, hampering timely diagnosis. Clinicians should consider possible voodoo poisoning in patients presenting with a history of drug use with neurological symptoms, and they should conduct follow-up arterial blood gases, electrolytes and ECG as voodoo may contain potentially fatal psychoactive substances.

## Introduction

The rapid emergence of new psychoactive substances (NPSs) into the illicit drug market is a major public health problem worldwide [1]. The European Union Early Warning System has reported the emergence of 620 NPSs over the last decade, including 101 during 2014 alone [2]. These NPSs include synthetic cannabinoids, cathinones, phenethylamines, tryptamines, piperazines, opioids, and plant-based substances among others [3]. Further, these substances are widely available and usually marketed as “legal highs” with no information to users about their types, safe dosing or potential adverse effects [4–6].

These NPSs differ widely in chemistry, so patients present with divergent symptoms and signs including agitation, palpitation, chest pain, seizures, and cardiotoxicity [7, 8].

In Egypt, the General Secretariat of Mental Health and Addiction Treatment (GSMHAT), Ministry Of Health (MOH) reported that the rates of substance use are steadily rising over time and young adults were the most vulnerable age group among substance users. The prevalence rate of NPSs use in Egypt is severely underestimated [9–11].The use of these novel substances, carrying street name known as Voodoo has recently spread among youth in Egypt. Substances being sold as “Voodoo” are usually packaged and sold as herbal incense for smoking. It was proved to contain heterogeneous mixtures of psychoactive substances such as synthetic cannabinoids, amphetamine, tramadol, methadone, MDA, benzodiazepines, morphine derivatives, and penitrem A (a neurotoxin). These NPSs are usually made in clandestine laboratories with substantial variation in the types and concentrations of chemical constituents and adulterants, further contributing to the variation in clinical presentation [12].

The Poison Control Center of Ain Shams University Hospitals (PCC-ASUH) is the first established poison center in Egypt and one of the main referral poison centers in the country. According to hospital records, 11 cases of voodoo intoxication were admitted to the ED by the end of 2015, of which two led to death. During 2016, the center received 75 cases of voodoo toxicity requiring emergency medical consultation, of which four resulted in death [13]. Despite this clinically significant fatality rate, there have been few studies on the acute toxic effects associated with voodoo use, so the manifestations predictive of hospitalization and death are still unknown. A more precise clinical description is critical both for improved care and to facilitate better regulatory and legal control. This study describes the acute toxic effects of voodoo in patients presentedto the PCC-ASUH during the year of 2017.

## Methodology

### Study design

This retrospective study design included all records of voodoo intoxicated cases presented to the PCC-ASUH during 2017.

### Patients

This study included all files of voodoo intoxicated patients presented to the PCC-ASUH from 1 January 2017 to 31 December 2017. The patient’s selection was based on presentation with a definite history of voodoo smoking (reported either by the patient or a witnesses in comatose cases) in addition to clinical manifestations of acute toxicity as evaluated by a clinical toxicologist such as agitation, seizures, coma, chest pain, vomiting or any other toxic symptom. We excluded files with insufficient information or an unclear diagnosis. Files of patients with a previously known cardiovascular, renal, or other medical condition that could contribute to the measured parameters were also excluded.

### Data collection

Data were collected from the medical records and extracted according to a predesigned data collection sheet. Data collected included age, sex, and intoxication history (the substance used as reported by the patient or witnesses, co-administration of other illicit substances, and delay time). Delay time was defined as the time between drug administration and ED presentation [14]. Additional recorded data included presented symptoms, clinical data (vital signs and results of skin, pupil, cardiovascular, neurological, and respiratory examinations), and laboratory findings (arterial blood gases, serum electrolytes, blood glucose level, total leukocyte count, serum aspartate aminotransferase (AST), serum alanine aminotransferase (ALT) levels, creatinine, urea, cardiac enzymes and total creatine phosphokinase (CPK)). Blood samples for routine investigations were obtained during initial patient presentation in the ED. Normal reference values of vital data are from Rees et al. [15].

The data collection sheet also included the results for urine screening for other illicit substances such as cannabis, tramadol, opiates, benzodiazepines, and amphetamine. These substances were detected using immunoassay techniques and confirmed by high-performance liquid chromatography (HPLC) and gas chromatography-mass spectrometry (GC-MS).

We also included the results of standard 12-lead ECG performed on all patients during the initial ED visit. ECG analysis included the heart rate, rhythm, ST/T abnormalities, conduction defects, and PR and QT intervals. The QT interval was corrected (QTc) according to the formula of Bazett [16], QTc = QT / √RR, which adjusts for heart rate by dividing the measured QT by the square root of the RR interval.

All patients were managed according to PCC-ASUH protocols for acute toxicity, starting with airway and oxygen maintenance, and if necessary endotracheal intubation and ventilatory support. These emergency measures were followed by symptomatic and supportive treatments as well as naloxone administration as an antidote in case of opioid toxidrome. Other treatments were administered as needed according to the clinical condition of the patient. Patient outcome was recorded as discharged after observation, admission to an inpatient unit, intensive care unit (ICU) admission, or death.

### Statistical analysis

The collected data were coded, tabulated, and statistically analyzed using IBM SPSS statistics (Statistical Package for Social Sciences) software version 22.0, IBM Corp., Chicago, USA, 2013. Quantitative normally distributed data described as mean ± SD (standard deviation) after testing for normality using Shapiro-Wilk test, then compared using ANOVA test. Qualitative data described as number and percentage and compared using Chi square test and Fisher’s Exact test for variables with small expected numbers. The level of significance was taken at P value < 0.05 was significant, otherwise was non-significant.

Diagnostic characteristics was calculated as follows:

- Sensitivity = (True positive test / Total positive golden) x 100.
- Specificity = (True negative test / Total negative golden) x 100.
- Diagnostic accuracy = ([True positive test + True negative test] / Total cases) x 100.
- Youden’s index = sensitivity + specificity – 1.
- Predictive positive value = (True positive test / Total positive test) x 100.
- Predictive negative value = (True negative test / Total negative test) x 100.

## Results

This retrospective study included 71 voodoo-intoxicated patients who presented to the PCC-ASUH from 1 January 2017 to 31 December 2017. The mean age of studied patients was 25.19 ± 9.54 years (range: 15–50 years), with the majority 15–24 years of age (59.1 %) or 25–34 years (23.9 %), while those older than 45 years represented only 5.6 % of all cases. The vast majority of cases were male (97.2 %) and average time between ingestion and presentation was 4.1 ± 2.7 h (range: 1–12 h).

Pulse was normal in more than half of cases (54.9 %), while tachycardia was observed in 22 cases (31 %) and bradycardia in 8 cases (11.1 %). More than half of patients also exhibited normal blood pressure (59 %), while 9 were hypotensive (12.6 %) and 20 were hypertensive (28.1 %). Respiratory rate was normal in 59.1 % of cases while 19 cases (26.7 %) were tachypneic and 10 cases (14.1 %) bradypneic.

Table 1 shows the clinical characteristics of voodoo-intoxicated patients. Table 2 summarizes laboratory findings and corresponding frequencies among voodoo-intoxicated patients.

**Table 1:** Clinical characteristics of voodoo-intoxicated patients

Clinical characteristics	Number (71)	Frequency (%)
**Neurological**		
Agitation	29	40.8
Coma	22	31
Seizures	10	14.1
Drowsiness	9	12.6
Hallucinations	7	9.8
**Cardiovascular**		
Palpitation	28	39.4
Chest pain	26	36.6
Cardiac arrest	1	1.4
**Pupil size**		
Miosis	11	15.6
Mydriasis	4	5.6
**Respiratory**		
Dyspnea	18	35..5
Cyanosis	11	15.4
Respiratory failure	11	15.4
Pulmonary edema	2	2.8
**Gastrointestinal**		
Vomiting	22	31
Epigastric pain	13	18.3

**Table 2:** Laboratory findings among voodoo-intoxicated patients

Laboratory data		Number (71)		Percentage (%)
**Arterial blood gases**	Normal	23		32.4
	Respiratory acidosis	39		54.9
	Metabolic acidosis	7		9.8
	Mixed metabolic and respiratory acidosis	2		2.8
**Glucose**	Normal	46		64.7
	Hyperglycemia	20		28.1
	Hypoglycemia	5		7.2
**Sodium**	Normal	58	81.6	
	Hyponatremia	13	18.3	
**Potassium**	Normal	47		66.2
	Hypokalemia	24		33.8
**Urea**	Normal	40		56.3
	Increased	31		43.6
**Creatinine**	Normal	64	90.1	
	Increased	7	9.8	
**AST**	Normal	62		87.3
	Increased	9		12.6
**ALT**	Normal	65		91.5
	Increased	4		5.6
**Total CPK**	Normal	60		84.5
	Increased	11		15.5
**CK-MB**	Normal	58	81.6	
	Increased	13	18.3	
**Troponin**	Normal	58	81.6	
	Increased	13	18.3	
**Total leukocytic count**	Normal	52	73.2	
	Increased	19	26.7	

AST: aspartate aminotransferase, ALT: alanine aminotransferase, CPK: creatine phosphokinase, CK-MB: creatine kinase- myocardial band

Based on urine screens, more than half of the studied patients (53.5 %) co-administered other illicit substances, including cannabis (12 cases, 17 %), tramadol (10 cases, 14.1 %), opiates (5 cases, 7 %), benzodiazepine (4 cases, 5.6 %), and amphetamine (2 cases, 2.8 %).The most common ECG finding was sinus tachycardia (31 %) followed by QT prolongation (15.4 %), sinus bradycardia (14 %), inverted T wave (11.2 %) and ST elevation (5.6 %).

Nine cases (12.4 %) required endotracheal intubation and 7 (9.8 %) required mechanical ventilation, while the majority received only supportive and symptomatic treatments. Gut decontamination by gastric lavage was not required as voodoo was consumed through inhalation in all cases. Naloxone was administered as an antidote in 2 patients (2.8 %) presented with opioid toxidrome. Other treatments administered according to patient condition included O_2_ therapy (21 %), diazepam (41 %), inotropics (4.5 %), H2 blockers (18.5 %), and IV fluids.

Table 3 shows the patient outcomes. Two patients died (2.8 %), both were males who inhaled voodoo alone as documented by negative screening for other illicit substances. One patient was suddenly arrested two hours from admission (ECG showed MI on admission) and the other patient died on the second day of admission from respiratory failure. Both patients had no past history of any chronic disease.

**Table 3:** Patient outcomes

Outcome	Number (71)	Frequency (%)
**Observation and discharge**	23	32.4
**Ward admission**	11	15.5
**ICU admission**	37	52.1
**Endotracheal intubation**	9	12.6
**Mechanical ventilation**	7	9.8
**Duration of hospital stay**		
**<6 hours**	23	32.4
**1–2 days**	38	53.5
**>2 days**	10	14.1
**Recovery**	69	97.2
**Death**	2	2.8

ICU: intensive care unit

Comparison was done between patients who used voodoo only and those who used it with other illicit substances as regards demographics, substance characteristics, clinical presentation, laboratory and ECG findings, we found that there was no significant differences except for tachycardia that was significantly higher in patients who used voodoo only than those who used voodoo with other illicit substances.

To account for differences between patients who observed in ER then discharged, those admitted to wards and ICU, the analysis adjusted for demographics, substance characteristics and clinical presentation (Table 4). This analysis revealed that there was no statistical significant difference between ER patients and patients who need hospitalization (ward or ICU) as regards sex, type of substances and delay time. Most of hospitalized patients aged between 20 and 39 years old. A statistical significant association was found between hospitalization and presence of bradycardia, tachypnea, coma and seizures among studied cases. Moreover, there was a significant association between ICU admission and presence of tachycardia, hypotension, bradypnea, shock and cyanosis among studied cases. Table 5 shows clinical variables in predicting hospitalization (ward or ICU) against ICU admission. The current study revealed that bradycardia, tachypnea, coma and seizures had high specificity& positive predictive value and lower other characteristics in predicting hospitalization (ward or ICU), yet presence of any of them had high different characteristics. In predicting ICU admission, we found that tachycardia, hypotension, bradypnea, shock and cyanosis had high specificity & positive predictive value and lower other characteristics, however presence of any of them elevated low characteristics to be moderate (Table 5).

**Table 4:** Demographics, substance characteristics and clinical presentation of the ER patients compared with ward admission group and the ICU admission group

Variables		ER observation (N = 23)	Ward admission (N = 11)	ICU admission (N = 37)	p-value
**Demographics and substance characteristics**					
**Age (years), Mean ± SD**		25.0 ± 11.2	27.3 ± 5.3	24.7 ± 9.5	^a^0.733
**Age categories (years)**	**< 20**	11 (47.8 %)	0 (0.0 %)	13 (35.1 %)	^c^ 0.014^d^
	**20 − 39**	9 (39.1 %)	11 (100.0 %)	20 (54.1 %)	
	**40 − 50**	3 (13.0 %)	0 (0.0 %)	4 (10.8 %)	
**Sex**	**Male**	23 (100.0 %)	11 (100.0 %)	35 (94.6 %)	^c^ 0.658
	**Female**	0 (0.0 %)	0 (0.0 %)	2 (5.4 %)	
**Type of substance**	**Mixed substances**	12 (52.2 %)	8 (72.7 %)	19 (51.4 %)	^b^0.434
	**Voodoo only**	11 (47.8 %)	3 (27.3 %)	18 (48.6 %)	
**Delay time (hours)**	**1 − 2**	11 (47.8 %)	2 (18.2 %)	14 (37.8 %)	^c^ 0.170
	**3**	8 (34.8 %)	3 (27.3 %)	8 (21.6 %)	
	**≥ 4.0**	4 (17.4 %)	6 (54.5 %)	15 (40.5 %)	
**Clinical presentation**					
**Tachycardia**		2 (8.7 %)	0 (0.0 %)	**20 (54.1 %)**	^b^<0.001^d^
**Bradycardia**		0 (0.0 %)	**2 (18.2 %)**	**8 (21.6 %)**	^c^ 0.037^d^
**Hypertension**		1 (4.3 %)	0 (0.0 %)	7 (18.9 %)	^c^ 0.114
**Hypotension**		0 (0.0 %)	0 (0.0 %)	**9 (24.3 %)**	^c^ 0.009
**Tachypnea**		0 (0.0 %)	**5 (45.5 %)**	**14 (37.8 %)**	^b^0.002^d^
**Bradypnea**		0 (0.0 %)	0 (0.0 %)	**10 (27.0 %)**	^c^ 0.004
**Fever**		0 (0.0 %)	0 (0.0 %)	2 (5.4 %)	^c^ 0.658
**Agitation**		3 (13.0 %)	2 (18.2 %)	13 (35.1 %)	^b^0.135
**Coma**		1 (4.3 %)	**5 (45.5 %)**	**22 (59.5 %)**	^b^<0.001^d^
**Seizures**		0 (0.0 %)	**2 (18.2 %)**	**8 (21.6 %)**	^c^ 0.037^d^
**Drowsiness**		4 (17.4 %)	2 (18.2 %)	3 (8.1 %)	^c^ 0.438
**Hallucination**		3 (13.0 %)	1 (9.1 %)	3 (8.1 %)	^c^ 0.862
**Hyporeflexia**		0 (0.0 %)	0 (0.0 %)	5 (13.5 %)	^c^ 0.133
**Miosis**		2 (8.7 %)	2 (18.2 %)	7 (18.9 %)	^c^ 0.599
**Mydriasis**		0 (0.0 %)	0 (0.0 %)	4 (10.8 %)	^c^ 0.275
**Chest pain**		11 (47.8 %)	4 (36.4 %)	11 (29.7 %)	^b^0.386
**Cardiac arrest**		0 (0.0 %)	0 (0.0 %)	2 (5.4 %)	^c^ 0.658
**Shock**		0 (0.0 %)	0 (0.0 %)	**9 (24.3 %)**	^c^ 0.009^d^
**Dyspnea**		**15 (65.2 %)**	**5 (45.5 %)**	**9 (24.3 %)**	^b^0.007^d^
**Cyanosis**		0 (0.0 %)	0 (0.0 %)	**11 (29.7 %)**	0.002^d^
**Vomiting**		6 (26.1 %)	4 (36.4 %)	12 (32.4 %)	^c^ 0.801
**Epigastric pain**		3 (13.0 %)	3 (27.3 %)	7 (18.9 %)	^c^ 0.586

ER: Emergency room, ICU: Intensive care unit. Data presents as number (percent) unless mentioned otherwise. ^a^Independent t-test. ^b^Chi square test. ^c^Fishers Exact test. ^d^Significant (< 0.050

**Table 5:** Clinical variables predicting hospitalization (ward or ICU) against ICU admission among voodoo intoxicated patients

Variables	Sensitivity	Specificity	Diagnostic accuracy	Youden’s index	Positive predictive value	Negative predictive value
**Hospitalization (ward or ICU)**						
**Bradycardia**	20.8 %	100 %	46.5 %	20.8 %	100 %	37.7 %
**Tachypnea**	39.6 %	100 %	59.2 %	39.6 %	100 %	44.2 %
**Coma**	56.3 %	95.7 %	69.0 %	51.9 %	96.4 %	51.2 %
**Seizures**	20.8 %	100 %	46.5 %	20.8 %	100 %	37.7 %
**Any of them**	83.3 %	95.7 %	87.3 %	79.0 %	97.6 %	73.3 %
**ICU admission**						
**Tachycardia**	54.1 %	94.1 %	73.2 %	48.2 %	90.9 %	65.3 %
**Hypotension**	24.3 %	100 %	60.6 %	24.3 %	100 %	54.8 %
**Bradypnea**	27.0 %	100 %	62.0 %	27.0 %	100 %	55.7 %
**Shock**	24.3 %	100 %	60.6 %	24.3 %	100 %	54.8 %
**Cyanosis**	29.7 %	100 %	63.4 %	29.7 %	100 %	56.7 %
**Any of them**	67.6 %	94.1 %	80.3 %	61.7 %	92.6 %	72.7 %

ICU: Intensive care unit

## Discussion

As a relatively new and chemically heterogeneous psychoactive substance, voodoo is difficult to identify by blood or urine drug screening and presents with unpredictable manifestations. Therefore, clinicians should use clinical history, routine laboratory parameters, and toxidromes to evaluate voodoo-intoxicated patients. In this study, we describe the clinical profile of voodoo intoxication by retrospectively reviewing the physical and biochemical parameters routinely assessed in the ED to further assist clinicians in diagnosis and treatment.

NPS use in the current study was found commonly among adolescents and young adults, consistent with previous studies on the demographic of people who use voodoo in Egypt and Croatia [5, 17]. Almost all our patients were male, consistent with a previous study in Egypt [9] reporting that females accounted for only 2.2 % of people who use voodoo. Eaton et al. [18] posited that males manifest psychopathology externally, such as through substance use and aggression, while females are more likely to manifest such problems internally as anxiety and depression.

Given the heterogeneous mix of components, little is known about the pharmacological actions of voodoo, but most patients examined in this cohort exhibited signs of sympathomimetic syndrome, consistent with previous reports documenting sympathomimetic toxidrome among patients with NPS toxicity, including cardiovascular effects such as palpitation, chest pain, tachycardia, and hypertension, in addition to neuropsychiatric manifestations such as confusion, agitation, hallucinations, and seizures [19–22]. This toxidrome may be caused by stimulants like amphetamine and MDA that have been found in voodoo samples from Egypt [12]. Alternatively, the agitation and hallucinations observed in many of our study patients may be explained by the effect of synthetic cannabinoids [12, 23].

Opioid toxidrome was also found in some of our patients, which manifested as coma, respiratory depression, and/or miosis. This toxidrome could be attributed to the effects of tramadol [12, 24]. Seizures observed in a minority of patients (14.1 %) may also result from tramadol-induced sympathomimetic toxidrome or the presence of penitrem A, a powerful neurotoxin [12].

Arterial blood gas analysis revealed respiratory acidosis in most studied patients, which could be explained by the depressant effect of either opioids or synthetic cannabinoids on respiration [25, 26] as these substances were found in Egyptian samples of voodoo by Hussien et al. [12].

Hyperglycemia found in the current study may be explained by respiratory depression and ensuing cerebral vasodilatation leading to enhanced glucose entry into the brain [27]. Conversely, hypoglycemia observed in a small number of our patients is consistent with Sanli et al. [28], who reported that some psychoactive substances may decrease blood glucose levels by suppressing gluconeogenesis, especially among patients with poor nutrition. Hyponatremia observed in our study may be explained by antidiuretic hormone release triggered by serotonergic pathway activation from sympathomimetic poisoning [29]. In contrast to our study, Sanli et al. [28] reported a significant increase in serum sodium among studied patients. Hypokalemia observed in some of our patients may also be caused by beta receptor activation associated with sympathomimetic toxicity [30]. Taskiran and Taskiran & Mutluay [31] reported hypokalemia in people who use synthetic cannabinoids, which were also found in voodoo [11].

. The increase in serum urea may be explained by dehydration secondary to vomiting, which was observed in some cases and can result in increased urea reabsorption by the kidney [32]. Additionally, Sanli et al. [28] speculated that nutritional factors such as low protein intake among people who use psychoactive substances may contribute to elevated serum urea. In addition, renal injury may also contribute to these abnormalities as sympathomimetic poisoning causes generalized vasoconstriction resulting in renal ischemia [33]. Furthermore, opioids found in voodoo may cause direct renal injury from glomerular immunoglobulin and amyloid deposition [34, 35].

Most of our study patients had normal AST and ALT, suggesting the absence of liver damage, but small subpopulations did present with elevated AST or ALT, consistent with rare cases of acute liver injury cause by sympathomimetics [36]. Núñez et al. [37] reported that sympathomimetic hepatotoxic effects vary from asymptomatic elevation of liver enzymes to acute liver failure requiring liver transplantation. Total CPK was normal in the majority of study patients but was elevated in 15.5 %. This result is in accordance with Rahimi et al. [38], who reported rhabdomyolysis as a rare complication of narcotics intoxication. On the other hand, rhabdomyolysis with high CPK level has been reported as a common complication of illicit drugs toxicity, in some cases secondary to myonecrosis resulting from prolonged coma and seizures [39, 40].

The cardiac enzymes CK-MB and troponin I were increased in some of studied patients, possibly due to the sympathomimetic effects of the voodoo components amphetamine, MDA, and synthetic cannabinoids, which are documented to promote cardiac toxicity leading to heart failure syndromes [12, 41]. The cardiotoxicity observed in some of our patients may be attributed either to voodoo constituents or the concomitant use of other illicit drugs. Fujita et al. [42] reported that the combined use of two psychoactive drugs with different pharmacological actions could lead to synergistic lethal cardiac consequences.

Leukocytosis noticed in some of patients in our study were consistent with Logan et al. [43]. Richards et al. [44] explained this leukocytosis as resulting from catecholamine release associated with sympathomimetic toxidrome.

Toxicological screening also revealed a high prevalence of illicit drug co-administration with voodoo, such as tramadol, cannabis, amphetamine, opiate, and benzodiazepines. This is in accordance with EMCDDA study findings [45] that most people who use NPS are commonly use other drugs with it and that a given NPS is rarely the primary illicit drug used; rather, it is usually taken when the preferred illicit substance is unavailable or to enhance the “high” of the preferred substance.

Sinus tachycardia was the most common ECG finding in voodoo intoxicated patients and it may be attributed to sympathomimetic toxidrome while sinus bradycardia that was noticed in the minority of studied patients may be attributed to opioid toxidrome. The ST-segment elevation found in our patients may be attributable to myocardial ischemia (MI) as Mir et al. [46] and Orsini et al. [47] described ST-segment elevation due to MI in synthetic cannabinoid-intoxicated patients. Indeed, several studies have reported acute MI from synthetic cannabinoids use [48–50]. Additionally, Backmund et al. [50] and Ioseliani et al. [51] reported MI due to use of methadone, which has been reported as a voodoo constituent [12]. Finally, while Castaneto et al. [52] reported that ECG changes indicative of MI were rare in people who use synthetic cannabinoid, it could be precipitated by other symptoms observed in our patients, such as tachycardia, hypertension, and metabolic disturbances.

More than two thirds of cases in our study required hospitalization. Khalifa and Lashin [53] suggested that predicting ICU admission through clinical parameters on admission might help physicians to identify patients at high risk for early monitoring and intensive treatment for a better outcome. Our study revealed that cases of acute voodoo poisoning who presented with any of bradycardia, tachypnea, coma and seizures required hospitalization (ward or ICU) and cases with any of tachycardia, hypotension, bradypnea, shock and cyanosis almost required ICU admission.

Voodoo toxicity has no consistent presentation due to the variation in constituents. Nonetheless, clinicians should consider voodoo poisoning in patients presenting with a history of recreational drug use or addiction and neurological symptoms such as agitation, hallucinations, and/or disturbed conscious level. Moreover, clinicians should check arterial blood gases, serum glucose, serum electrolytes, and total leukocytic counts, and consider follow-up ECG with close monitoring in the ICU as voodoo may contain harmful and potentially fatal psychoactive substances.

### Limitation of the study

The diagnosis of voodoo intoxication was based mainly on information provided by the patient or witnesses in comatose cases as there is no analytical test for confirmation. Subsequent studies should aim to obtain raw samples of the consumed drug from patients, witnesses, or appropriate authorities to establish associations between contents and symptoms. In addition, one of the major limitations of this study was the bias of the retrospective design as we depended mainly on the records of voodoo intoxicated patients who presented to the PCC-ASUH during year 2017.

## Conclusion and recommendations

Toxicity from voodoo substance can manifest in many ways and in rare cases result in death. The most common clinical manifestations are neurological such as agitation, hallucinations, and/or disturbed consciousness. Respiratory acidosis is a common laboratory abnormality and sinus tachycardia is a common ECG finding. These symptoms may be dependent on the specific composition of the individual voodoo sample. New analytical methods are urgently needed to detect and quantify the components of this new psychoactive substance in biological fluids. Moreover, awareness of the harmful effects of voodoo use should be promoted, especially among young people and also awareness among physicians about the initial clinical findings of acute voodoo poising to seek the appropriate level of medical care and better outcome.

## Data Availability

All data generated or analyzed during this study are included in this article.

## Abbreviations

ALT: Alanine aminotransferase
AST: Aspartate aminotransferase
CK-MB: Creatine kinase-MB
CPK: Creatine phosphokinase
ECG: Electrocardiography
ED: Emergency department
EMCDDA: European Monitoring Centre for Drugs and Drug Addiction
GC-MS: Gas chromatography-mass spectrometry
GSMHAT: General Secretariat of Mental Health and Addiction Treatment
h: Hour
HPLC: High-performance liquid chromatography
ICU: Intensive care unit.
MDA: 3,4-Methylenedioxyamphetamine
MI: Myocardial infarction
MOH: Ministry Of Health
mm: millimeter
NPSs: New psychoactive substances
PCC-ASUH: Poison Control Center of Ain Shams University Hospitals
